# In Utero Molecular-Targeted Drug Therapies: Translational Principles, Pharmacologic Considerations, and Emerging Clinical Applications

**DOI:** 10.3390/jcm15134960

**Published:** 2026-06-25

**Authors:** Akihiro Hasegawa, Ehsan Rojhani, Ahmed Hashem Fathallah, Rodrigo Ruano, Alireza Abdollah Shamshirsaz

**Affiliations:** 1 Fetal Care and Surgery Center, Division of Fetal Medicine and Surgery, Boston Children’s Hospital, Harvard Medical School, Boston, MA 02115, USA; a_hasegawa1011@yahoo.co.jp (A.H.); ehsan.rojhani@austin.utexas.edu (E.R.); 2 Faculty of Medicine, Minia University, Minia 2431436, Egypt; dr.ahmedhashem12@gmail.com; 3 Division of Maternal-Fetal Medicine, Department of Obstetrics, Gynecology & Reproductive Sciences, University of Miami, Miami, FL 33136, USA; rodrigoruano@hotmail.com; 4 Comprehensive Fetal Care Center at Dell Children’s Medical Center, Dell Medical School, The University of Texas at Austin, Austin, TX 78723, USA

**Keywords:** fetal therapy, in utero therapy, molecular-targeted therapy, prenatal pharmacotherapy, precision medicine, review

## Abstract

Advances in fetal diagnosis and molecular medicine have opened new opportunities for in utero molecular-targeted drug therapy, shifting fetal treatment from purely procedural interventions toward pharmacologic strategies that address disease mechanisms before irreversible organ damage occurs. In this review, we highlight recent advances in in utero drug therapy, focusing on molecular-targeted approaches with emerging clinical or trial-level evidence. Early clinical experience and ongoing trials have demonstrated the feasibility of achieving therapeutically relevant fetal drug exposure, although the strength of evidence varies considerably across therapeutic classes. However, significant challenges remain, including optimization of fetal drug delivery, characterization of fetal pharmacokinetics and pharmacodynamics, long-term safety assessment, and ethical considerations. The current evidence base ranges from single case reports to ongoing Phase 3 clinical trials, underscoring both the promise of prenatal molecular therapeutics and the need for further prospective evaluation. Continued integration of fetal imaging, genomics, ethics and pharmacology will be essential to advance safe and effective prenatal precision therapies.

## 1. Introduction

Improvements in prenatal imaging and genetic diagnostics now allow fetal abnormalities to be detected at increasingly earlier gestational stages, thereby expanding the therapeutic window for intervention before irreversible injury occurs [1]. Over the past several decades, the field of fetal therapy has evolved from pioneering intrauterine transfusion to increasingly sophisticated approaches, including fetoscopic procedures, transplacental pharmacotherapy, stem cell transplantation, and emerging gene- and molecular-based strategies aimed at modifying disease trajectories before birth [1,2,3]. Several unique aspects of fetal physiology provide a compelling rationale for prenatal intervention. These include the relative immaturity of the fetal immune system, the potential to prevent or reverse pathology at an early developmental stage, the presence of accessible niches within fetal organs, and the smaller fetal body mass-to-volume ratio, which may reduce therapeutic dosing requirements [2,4,5].

Historically, pharmacologic treatment during pregnancy has been approached cautiously due to concerns regarding fetal teratogenicity, maternal safety, and adverse pregnancy outcomes [6,7,8]. However, with appropriate risk–benefit assessment and growing clinical experience, several medications are now routinely and safely used for fetal indications. Examples include antiarrhythmic agents for fetal tachyarrhythmias, antenatal corticosteroids for fetal lung maturation in threatened preterm birth, and immunomodulatory therapies for anti SS-A antibody-mediated fetal bradyarrhythmia [9,10]. In parallel, postnatal molecular therapies for congenital disorders have advanced substantially, including enzyme replacement therapy for lysosomal storage diseases, survival motor neuron (SMN)-targeted therapies for spinal muscular atrophy, and cystic fibrosis transmembrane conductance regulator (CFTR) modulator treatments for cystic fibrosis [11,12,13,14]. These innovations have significantly improved survival and quality of life for affected children and their families. Despite these advances, many congenital disorders—particularly monogenic, metabolic, and neuromuscular diseases—are characterized by progressive cellular injury that begins early in gestation and may become irreversible by the time postnatal therapy is initiated. Recognition that postnatal interventions may be insufficient to fully reverse established fetal damage has catalyzed growing interest in prenatal molecular-targeted therapy as a means to alter disease course at its earliest developmental stages [15,16,17].

Reflecting this shift, momentum in in utero molecular-targeted therapy has accelerated markedly in recent years, driven by advances in prenatal genomic diagnostics, the expansion of precision therapeutics, and increasing clinical experience with fetal pharmacologic intervention. This review summarizes the current landscape of clinical trials and emerging translational strategies, while also addressing the ethical, regulatory, and clinical challenges that define the evolving boundaries of fetal medicine in the era of prenatal precision therapy. This narrative review was based on literature identified through PubMed and ClinicalTrials.gov, including relevant clinical, translational, and preclinical studies published in English from database inception through May 2026.

## 2. Conceptual Framework for In Utero Molecular-Targeted Therapy

**Figure 1:**
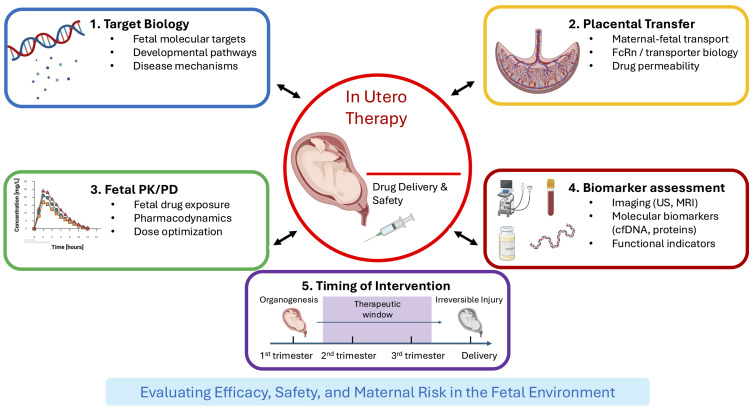
Translational framework for in utero molecular-targeted therapy. Development of effective prenatal molecular therapy requires integration of multiple biological and clinical domains. Key components include: (1) target biology, involving identification of fetal molecular targets and developmental disease pathways; (2) placental transfer, which determines maternal–fetal transport and fetal drug exposure; (3) fetal pharmacokinetics/pharmacodynamics (PK/PD), including fetal drug levels, biological activity, and dose optimization; (4) biomarker assessment, using imaging and molecular indicators to monitor disease severity and therapeutic response; and (5) timing of intervention, emphasizing treatment during developmental windows. The timeline illustrates progression through gestation from the first trimester (weeks 1–13), second trimester (weeks 14–27), and third trimester (weeks 28–40) to delivery. The shaded “therapeutic window” denotes the period during which prenatal intervention may provide the greatest benefit, after major organogenesis is largely complete, when fetal diagnosis becomes feasible, and before irreversible fetal injury occurs. Created in BioRender. Hasegawa, A. (2026) https://BioRender.com/XH29O3844C (accessed on 15 May 2026).

Before discussing specific therapeutic platforms, it is important to consider the fundamental principles that distinguish prenatal molecular-targeted therapies from conventional postnatal treatments. Successful prenatal intervention requires not only identification of an appropriate molecular target but also effective delivery of the therapeutic agent to the fetus while minimizing maternal and fetal risks. Understanding the available routes of fetal drug exposure provides a framework for interpreting the potential advantages, limitations, and clinical applications of these emerging therapies (Figure 1).

### 2.1. Approaches to the Fetus In Utero

In utero molecular-targeted therapies may be delivered through several routes, broadly categorized into indirect maternal administration and direct fetal access. Selection of the optimal delivery strategy requires careful consideration of fetal targeting, maternal safety, procedural risk, and the ability to achieve therapeutic concentrations within the fetal compartment.

Indirect fetal therapy is most commonly achieved through maternal systemic administration, either intravenously or orally, with subsequent drug transfer across the placenta. This transplacental approach is clinically established in the treatment of fetal arrhythmias, where maternal antiarrhythmic therapy can effectively restore fetal rhythm and improve outcomes [10]. In addition, preclinical studies have explored maternal delivery of enzyme replacement therapies, including intramuscular maternal administration as a potential strategy for fetal exposure [18,19].

The placenta serves as the critical interface between maternal and fetal circulations, functioning as a semi-permeable barrier that enables gas exchange, nutrient transport, and waste elimination, while also regulating fetal exposure to exogenous substances, including therapeutic agents. Structurally, the human placenta is lined by a continuous epithelial layer of multinucleated syncytiotrophoblasts, formed by fusion of cytotrophoblasts, which separates maternal blood from the fetal vascular compartment [20]. The apical membrane of the syncytiotrophoblast is directly bathed in maternal blood, whereas its basal membrane interfaces with fetal capillaries within the chorionic villi [21]. Because tight junctions prevent paracellular passage, placental drug transfer occurs primarily through passive diffusion and/or transporter-mediated mechanisms. Small, lipophilic molecules with molecular weights below ~500 Da may cross the placenta relatively efficiently via diffusion, whereas larger, charged, or protein-bound compounds often require active transport or may be excluded altogether. Key transporter families include ATP-binding cassette efflux transporters and solute carrier uptake transporters, which strongly influence fetal drug exposure [22]. Accordingly, maternal systemic delivery represents the least invasive route and is particularly attractive for small-molecule therapies with favorable placental permeability. However, placental transfer is highly variable, depending on gestational age, molecular size, lipophilicity, protein binding, and transporter affinity. These complexities necessitate careful pharmacokinetic modeling and may limit the feasibility of certain biologics, motivating the development of direct fetal delivery approaches.

Direct fetal therapy encompasses routes such as intravascular, intraperitoneal, and intra-amniotic delivery, typically performed under ultrasound guidance [23,24,25]. These approaches allow more precise dosing and higher fetal drug exposure but carry procedural risks, including preterm premature rupture of membranes, infection, fetal injury, and pregnancy loss. Preclinical studies have further explored organ-targeted delivery methods, including intrahepatic and intracranial administration of molecular therapeutics [26,27]. Such strategies highlight the expanding technical possibilities of fetal intervention but remain limited to experimental settings. Intra-amniotic administration has traditionally been viewed as an indirect fetal route, relying on fetal swallowing and gastrointestinal absorption. More recently, however, transamniotic strategies have gained attention as potential platforms for delivering stem cells, mRNA-based therapeutics, and recombinant proteins. Notably, emerging work suggests that certain agents introduced into the amniotic cavity may access the fetal circulation through specialized placental or membrane-associated transport pathways, sometimes conceptualized as a “placental gateway” mechanism [28,29,30].

Ultimately, the selection of delivery route must balance therapeutic efficacy with maternal and fetal safety. Continued advances in minimally invasive techniques, targeted delivery systems, and placental transport biology are expected to expand the feasibility and precision of direct fetal molecular therapies in the coming years.

### 2.2. Rationale for the In Utero Approach

The major theoretical advantages and disadvantages of in utero molecular-targeted therapies are summarized in Table 1. Key potential advantages include reduced therapeutic dose requirements, the possibility of immune tolerance induction, and the opportunity to intervene before irreversible organ damage occurs. Important limitations include maternal drug exposure, off-target fetal effects, challenges in dose optimization, ethical considerations, and limited long-term safety data.

First, the relatively small size of the fetus may substantially reduce the dose required per recipient weight. For example, the average fetal weight at 20 weeks’ gestation is approximately 300 g, compared with ~3.5 kg in a term newborn and ~70 kg in an adult (weight ratio ~1:12:233) [3]. This difference has important translational implications, particularly for biologic and pharmacologic therapies that require weight-based dosing. In principle, achieving therapeutic concentrations in utero may require markedly smaller quantities of therapeutic agents than would be needed after birth, potentially improving feasibility and reducing treatment costs. The magnitude of this advantage, however, may vary depending on the specific drug, placental transfer characteristics, and pharmacokinetic properties.

Second, in utero administration may promote immune tolerance to biologic therapeutics. A major limitation of postnatal ERT is the development of neutralizing anti-drug antibodies, which can reduce efficacy over time and complicate long-term disease management [31]. The fetal immune system is characterized by relative immaturity and a bias toward tolerance, which has profound implications for prenatal molecular therapy. Human fetal secondary lymphoid tissues contain a high proportion of CD4^+^CD25^+^FoxP3^+^ regulatory T-cells (Tregs), comprising approximately 13–22% of the CD4^+^ T-cell population [32]. These fetal Tregs contribute to immune regulation in utero, including the establishment of maternal microchimerism, by suppressing excessive immune responses to maternally derived cells crossing the placenta [33]. Collectively, this immunologic environment may allow sustained therapeutic activity with reduced immune neutralization. Clinical experience, however, remains limited. In the first reported case of prenatal enzyme replacement therapy for cross-reactive immunologic material Cross-Reactive Immunologic Material (CRIM)-negative infantile-onset Pompe disease, anti-drug antibodies developed after the third in utero infusion, and postnatal immune tolerance induction with rituximab, methotrexate, and intravenous immunoglobulin (IVIG) was still required, suggesting that in utero exposure alone may be insufficient to achieve durable immune tolerance in highly immunogenic conditions [34]. Thus, while fetal exposure may contribute to tolerance, in utero therapy alone may be insufficient to fully prevent antibody formation in highly immunogenic contexts. Similarly, in the setting of in utero hematopoietic stem cell transplantation, gestational timing appears critical. Earlier gestational intervention may be more favorable for tolerance induction, as the human adaptive immune system remains developmentally immature in early to mid-gestation, while peripheral fetal T-cell populations expand progressively during the second trimester [35]. Further investigation of fetal immune ontogeny will be essential as prenatal molecular trials expand.

Third, compared with postnatal intervention, fetal therapy offers the opportunity to interrupt disease pathogenesis before irreversible organ damage occurs. Many monogenic, metabolic, and neuromuscular disorders initiate cellular injury during mid-gestation or earlier, limiting the effectiveness of postnatal “rescue” strategies. Although human data remain sparse, emerging reports suggest the potential clinical impact of early molecular intervention. Prenatal risdiplam administration has been associated with the absence of postnatal symptoms in an infant at high risk for spinal muscular atrophy [36]. Likewise, in the Pompe disease case noted above, initiation of alglucosidase alfa in utero followed by continued postnatal therapy was associated with attenuation of cardiomyopathy and preserved early motor development [34]. Beyond genetic disorders, fetal pharmacologic therapy is already well established in certain acquired fetal conditions. For example, transplacental antiarrhythmic treatment for fetal tachyarrhythmia can prevent or reverse hydrops fetalis by restoring normal fetal heart rate, thereby improving perinatal outcomes [10]. These experiences highlight the broader principle that timely prenatal intervention may meaningfully alter fetal disease trajectories.

Despite these advantages, in utero therapy also carries important disadvantages and uncertainties. These include maternal drug exposure, off-target fetal effects, challenges in dose optimization, and ethical complexity surrounding interventions in a fetus who cannot provide consent [37]. From a maternal perspective, any pharmacologic or procedural intervention represents treatment in an otherwise healthy individual, necessitating particularly stringent safety thresholds [8]. Furthermore, long-term safety data are inherently limited, and unintended developmental effects may not become apparent until later in childhood or adulthood. Accordingly, careful risk–benefit assessment, rigorous translational evaluation, and long-term follow-up will be essential before widespread clinical adoption of prenatal molecular-targeted therapies.

## 3. Therapeutic Classes and Representative Case Studies

### 3.1. Enzyme Replacement Therapy for Lysosomal Storage Diseases

Lysosomal storage diseases (LSDs) are a group of monogenic conditions characterized by enzyme deficiencies that lead to the toxic accumulation of substrates within lysosomes, often resulting in severe, progressive multi-organ pathology [38]. While postnatal ERT has established itself as the standard of care for various LSDs, its efficacy is inherently limited by the timing of initiation and biodistribution constraints [39]. A major limitation of postnatal ERT is its inability to reverse pathology that accumulates during fetal development; for instance, in Mucopolysaccharidosis (MPS) VII and Infantile-Onset Pompe Disease (IOPD), manifestations such as non-immune hydrops fetalis and cardiomyopathy can result in fetal demise or irreversible organ damage prior to birth [17,34]. Standard recombinant enzymes administered systemically do not cross the blood–brain barrier (BBB), leaving the central nervous system (CNS) components of these diseases largely untreated [17,40]. In utero enzyme replacement therapy (IUERT) addresses these obstacles by introducing the enzyme when the fetal BBB is more permeable and by mitigating cellular damage during organogenesis [17].

The potential for IUERT to alter the natural history of severe LSDs was first reported in a case of a fetus with CRIM-negative IOPD [34]. The fetus received six in utero infusions of alglucosidase alfa starting at 24 weeks of gestation [34]. Unlike the two previously affected siblings who succumbed to cardiomyopathy and respiratory failure in infancy, the treated patient exhibited normal cardiac function at birth, age-appropriate motor milestones, and normal biomarkers (creatine kinase and glucose tetrasaccharide) through 13 months of life [17,34]. Moreover, placental histology revealed an absence of glycogen accumulation, suggesting that IUERT prevented substrate buildup in the fetal compartment [34].

The feasibility of this approach is currently being evaluated in the phase 1 PEARL (PrEnAtal Enzyme Replacement Therapy for Lysosomal Storage Diseases) clinical trial (NCT04532047) [17]. This trial enrolls fetuses diagnosed with specific early-onset LSDs, including MPS I, II, IVA, VI, and VII, IOPD, neuronopathic Gaucher disease, and Wolman disease, for which an FDA-approved recombinant enzyme is available [17,41]. The protocol involves weight-adjusted ERT administered via the umbilical vein every two to four weeks between 18 and 35 weeks of gestation [17].

Unlike transplacental pharmacotherapies, IUERT achieves fetal drug delivery through direct intravascular administration via the umbilical vein, thereby bypassing placental transfer. In the first reported case of CRIM-negative IOPD, fetal plasma enzyme activity and anti-drug antibody titers were serially monitored during treatment, demonstrating the feasibility of assessing fetal exposure and pharmacodynamic response in utero. Ongoing data from the PEARL trial may further inform optimal dosing strategies and fetal pharmacokinetics across different lysosomal storage disorders.

The development of high-titer anti-drug antibodies in CRIM-negative patients who lack endogenous protein can neutralize ERT and precipitate clinical decline [42,43]. A theoretical advantage of IUERT is the induction of immune tolerance by exposing the developing fetal immune system to the recombinant protein during the pre-immune stage [41]. In the case IOPD reported by Cohen, the patient developed transient low-titer anti-recombinant human acid alpha-glucosidase antibodies that resolved shortly with postnatal immune tolerance induction [34]. This aligns with preclinical data from MPS VII mouse models, where in utero administration of the enzyme resulted in immune tolerance to the recombinant protein even after repeated postnatal exposures [17,44].

IUERT seems to be a feasible intervention that may serve as a bridge to definitive postnatal therapy [17]. By preventing the onset of irreversible organ damage and inducing immune tolerance, IUERT represents a promising shift toward proactive fetal intervention [41]. However, current dosing strategies remain largely extrapolated from postnatal protocols and adjusted according to estimated fetal weight. Future research must focus on defining the long-term neurodevelopmental outcomes of in utero-treated individuals and expanding the eligibility to other metabolic conditions with prenatal onset.

### 3.2. CFTR Modulator Therapy for Cystic Fibrosis

Cystic fibrosis (CF) is a progressive multisystem genetic disorder historically associated with substantial morbidity and reduced life expectancy. The development of CF transmembrane conductance regulator (CFTR) modulators, particularly elexacaftor/tezacaftor/ivacaftor (ETI) therapies, has markedly improved survival and quality of life in individuals with responsive variants [14]. Recently, ETI has improved pulmonary function, nutritional status, and reproductive health, contributing to an increasing number of pregnancies among individuals with CF [45,46,47,48].

Although still limited, emerging pharmacokinetic and translational data indicate that ETI readily crosses the placenta and achieves biologically meaningful fetal exposure. Detectable concentrations of ETI components have been identified in cord blood, newborn plasma, and breast milk, in some cases at levels comparable to maternal circulation, supporting efficient transplacental transfer [46]. Owing to their lipophilic properties, ETI components are thought to cross the placenta and directly improve dysfunctional fetal CFTR activity by restoring chloride transport and fluid homeostasis in developing tissues, providing a biologically plausible explanation for the prenatal improvement of manifestations such as meconium ileus and bowel obstruction. Preclinical studies further suggest that fetal ETI exposure may influence developmental pathways. In a pregnant rat model, ETI resulted in widespread fetal tissue exposure without overt structural abnormalities, although transcriptomic changes were observed in the fetal thymus and cortex, including genes related to neuronal and muscle system development [49]. While no clear teratogenicity has been demonstrated at clinically relevant doses, these findings suggest that fetal pharmacodynamic effects may extend beyond simple drug transfer. Importantly, no human pregnancy data are currently available for vanzacaftor or deutivacaftor, and their fetal safety profiles remain uncertain.

Prenatal administration of ETI to carrier mothers has emerged as a potential strategy to treat fetal manifestations of CF, particularly meconium ileus (MI) [47,48,50]. Successful resolution of hyperechogenic bowel and prevention of MI has been documented in cases where ETI was initiated in the second or third trimester, with some fetuses showing complete resolution of bowel dilation on ultrasound before delivery [50]. Recent case reports have further substantiated the efficacy of maternal ETI therapy for fetal MI. In one report, a carrier mother with two previously affected pregnancies underwent fetal ultrasound at 23 weeks’ gestation that revealed dilated small-bowel loops consistent with MI. Maternal ETI was initiated at 26 weeks, and serial fetal magnetic resonance imaging demonstrated resolution of both MI and distal microcolon by 33 weeks. The infant was delivered at term with normal liver function, required no neonatal bowel surgery, and remained clinically well without surgical intervention at 12 months of age [51]. Similarly, another case report described a carrier mother in whom ultrasound at 21 weeks identified hyperechogenic bowel followed four weeks later by bowel dilation suggestive of MI. Maternal ETI was initiated at 31 weeks following diagnostic confirmation by amniocentesis. Weekly fetal surveillance demonstrated complete normalization before delivery at 39 weeks, and the infant was born without intestinal obstruction. Interestingly, fecal elastase initially measured 58.8 µg/g but normalized to 314 µg/g by day 21 of life, suggesting a potential therapeutic effect on exocrine pancreatic function [52]. In a recent cohort, resolution of bowel obstruction was observed as early as three days following ETI initiation, with 6 out of 8 curative cases achieving resolution within a median of 14 days [53]. Nonetheless, meconium ileus persistence has also been reported; in one case, where ETI was initiated at 27 weeks of gestation, necessitating emergency postnatal surgery [47]. This occurred despite relatively early treatment initiation, suggesting that factors beyond timing—including severity of obstruction, maternal body mass index affecting drug exposure, and variability in placental transfer—may influence therapeutic efficacy [47].

Unlike in MI, the ability of ETI to prevent exocrine pancreatic insufficiency (PI) and other structural damage when initiated in the second or third trimester is less certain [54]. In a study of two infants treated prenatally starting in the second/third trimester, both exhibited PI and signs of early CF lung disease despite treatment. These observations raise the possibility that pancreatic injury may occur earlier in fetal development than the window targeted by current protocols; however, this hypothesis is based on only two reported cases and requires prospective validation [54]. In contrast, infants exposed to modulators from conception have demonstrated pancreatic sufficiency at birth, although the available data remain limited [55,56]. Taken together, these findings suggest that earlier and sustained CFTR correction may influence organ preservation, but definitive conclusions regarding the optimal timing of prenatal intervention cannot yet be drawn.

The safety profile of in utero ETI exposure has thus far been generally reassuring, with no clear signal of major teratogenicity identified in prospective cohorts [57]. In a series of 58 prospective pregnancies, the rate of major congenital anomalies was 3.4%, which is consistent with the general population [57]. However, adverse events such as transient hepatotoxicity and rare respiratory distress have been noted, warranting careful neonatal monitoring [57,58]. Concerns regarding congenital cataracts have also emerged from preclinical rodent studies. Consistent with these findings, one case series reported bilateral congenital cataracts in 3 of 23 neonates exposed to ETI in utero, although the lesions were small and did not result in clinically significant visual impairment [45,58,59]. The potential effect of prenatal ETI exposure on the male reproductive tract remains uncertain. To date, preservation of the vas deferens has been documented in only two reported cases of male infants exposed to ETI throughout gestation [56]. Recent evidence suggests that third-trimester exposure alone is unlikely to prevent congenital bilateral absence of the vas deferens, as degeneration of the vas deferens is thought to begin during the second trimester [53]. Preclinical studies instead suggest that earlier fetal exposure may be required to protect the developing male reproductive tract [45,53]. Animal reproductive studies overall have demonstrated placental transfer of CFTR modulators without evidence of teratogenicity at human-equivalent therapeutic doses [60]. In humans, miscarriage rates among women maintained on ETI during pregnancy (8.9%) appear comparable to those in the general population, and reported congenital anomalies have generally been considered unrelated to ETI exposure or confounded by maternal comorbidities such as diabetes [61].

The maternal safety profile of ETI during pregnancy requires balancing potential but incompletely understood fetal risks against the known maternal risks associated with treatment discontinuation. Women who discontinue ETI because of pregnancy-related concerns frequently experience clinical deterioration, including severe pulmonary exacerbations and substantial declines in lung function necessitating treatment reinitiation [61]. Although complications such as gestational diabetes and preeclampsia have been reported in women continuing ETI during pregnancy, these events are generally considered unrelated to the medication, with isolated exceptions such as one reported case of cholecystitis that was considered possibly treatment-related [61]. Restoration of CFTR function with highly effective modulators may also increase fertility, potentially contributing to unplanned pregnancies and underscoring the importance of proactive reproductive counseling.

CFTR modulators are excreted into breast milk, as demonstrated in both animal lactation models and human pharmacokinetic studies [60]. Despite infant exposure through breastfeeding, a survey of 26 breastfed infants born to mothers continuing ETI postpartum reported no significant adverse outcomes [61]. The ongoing prospective MAYFLOWERS trial is designed to systematically characterize ETI pharmacokinetics in breast milk and infant plasma in order to refine evidence-based safety recommendations for this exposure scenario [60].

### 3.3. Risdiplam for Spinal Muscular Atrophy

Spinal muscular atrophy (SMA) is an autosomal recessive neuromuscular illness marked by the degeneration and loss of alpha motor neurons in the anterior horn of the spinal cord and brainstem motor nuclei, leading to progressive muscle weakness and respiratory insufficiency [15]. The rationale for pursuing in utero therapy for fetuses diagnosed with spinal muscular atrophy (SMA), in particular, those with two copies of survival of motor neuron 2 (SMN2) which predicts severe Type I disease, is based upon evidence that irreversible neuromuscular pathology begins during fetal development, establishing a therapeutic window that may close before birth [62,63,64]. Postnatal therapies initiated through newborn screening (NBS) have substantially improved outcomes but may not fully ameliorate disease burden in the most severe genotypes due to pre-existing damage sustained in utero [62,65]. The timing of NBS-based diagnosis introduces additional vulnerability. The median time to treatment through NBS is approximately 24–26 days of life; by this interval, half of infants with two SMN2 copies may already be overtly symptomatic, with substantial motor unit loss [62].

Previous research revealed delays in radial growth of motor axons and their subsequent ensheathment by Schwann cells in affected fetuses. These developmentally arrested, immature axons undergo precipitous degeneration immediately postnatally; in murine models, this loss occurs within the first two postnatal days [62]. Human autopsy studies also confirm diminished spinal motor neuron counts and immature axonal morphologies in neonates with severe SMA, substantiating that the disease process is active prenatally [62,66]. In the NURTURE trial, infants with two SMN2 copies treated presymptomatically achieved motor milestones, yet 16% still required respiratory intervention during acute illnesses, and some required gastrostomy tube placement [65]. Postnatal therapeutic efficacy is fundamentally constrained by the inability to reverse axonal injury established in utero [64].

To address these postnatal constraints and exploit the critical therapeutic window before it closes, interest has shifted toward small-molecule SMN2 splicing modifiers that can cross the placental barrier, with Risdiplam emerging as a leading candidate for maternal administration. Risdiplam distributes centrally and peripherally to increase functional survival motor neuron (SMN) protein levels [67]. Currently approved for postnatal use in patients aged two months and older (and recently from birth in some jurisdictions), the therapeutic window for severe SMA may also extend to the in utero period [15]. Prenatal intervention for SMA Type 0 and I could potentially prevent the motor neuron degeneration and developmental arrest that begin before birth and remain largely irreversible through neonatal treatment alone [15,68].

Risdiplam’s small molecular weight and oral bioavailability theoretically allow for transplacental transfer from mother to fetus [15]. The first reported prenatal use of risdiplam was recently described in a case report involving a fetus diagnosed with SMA with two copies of SMN2 and a family history of severe Type I SMA. In this case, the mother received 5 mg of oral risdiplam daily starting at 32 weeks and 5 days of gestation until delivery [36]. Pharmacokinetic analysis at delivery demonstrated transplacental passage, with risdiplam concentrations in the umbilical vein reaching approximately 69% of maternal plasma levels and 33% in amniotic fluid. Pharmacodynamic analysis demonstrated higher SMN protein levels and lower neurofilament levels in the infant, suggesting biological target engagement during the final weeks of gestation. At 30 months of age, the child, who continued postnatal risdiplam, remained free of clinical manifestations of SMA and demonstrated age-appropriate muscle and peripheral nerve development [36]. However, these findings are derived from a single case report, and no systematic human prenatal pharmacokinetic, safety, or efficacy data currently exist. Consequently, conclusions regarding the neuroprotective efficacy and long-term safety of prenatal risdiplam remain preliminary and require confirmation through additional clinical experience and prospective studies.

The safety of risdiplam during pregnancy remains an area of scrutiny due to preclinical signals of embryofetal toxicity. Animal studies in rats and rabbits indicated that risdiplam crosses the placenta and may cause teratogenic effects, although specific clinical data in human pregnancy are sparse [67]. Ophthalmologic safety is yet another concern for risdiplam due to retinal toxicity observed in preclinical primate models at high doses. Fortunately, extensive monitoring of 245 patients across the FIREFISH, SUNFISH, and JEWELFISH trials revealed no evidence of retinal toxicity or functional visual changes in humans exposed to therapeutic doses [69].

### 3.4. FcRn Blockade with Nipocalimab for HDFN and FNAIT

Hemolytic disease of the fetus and newborn (HDFN) and fetal/neonatal alloimmune thrombocytopenia (FNAIT) are prototypical alloimmune disorders of pregnancy in which maternal immunoglobulin G (IgG) antibodies cross the placenta and injure fetal cells [70,71]. In HDFN, maternal alloantibodies directed against fetal erythrocyte antigens cause progressive hemolysis and fetal anemia, which may lead to hydrops fetalis, preterm delivery, or intrauterine demise [72]. In FNAIT, maternal antibodies against fetal platelet antigens cause thrombocytopenia and confer a substantial risk of fetal intracranial hemorrhage (ICH), a complication that frequently occurs in utero and is associated with high mortality and long-term neurologic morbidity [73]. Severe ICH events occur before 28 weeks’ gestation, often prior to clinical detection, underscoring the need for preventive strategies [74]. Despite advances in prenatal diagnosis, current management remains largely reactive. HDFN is monitored with middle cerebral artery Doppler and treated with intrauterine transfusion (IUT), whereas FNAIT relies on maternal IVIG, with or without corticosteroids. These approaches are indirect, variably effective, and do not address the underlying mechanism of maternal–fetal antibody transfer, leaving a clear need for mechanism-based prenatal therapies [71,72,75,76,77].

The neonatal Fc receptor (FcRn) plays a central role by mediating both placental IgG transport and systemic IgG homeostasis [78]. Expressed in the syncytiotrophoblast, FcRn enables receptor-mediated transcytosis of IgG from maternal to fetal circulation. At the same time, FcRn recycles IgG within endothelial and hematopoietic cells, prolonging its half-life [70]. While this system is essential for passive neonatal immunity, it also facilitates transfer of pathogenic alloantibodies. IgG transfer increases with gestational age, leading to greater fetal exposure later in pregnancy. These features position FcRn as a key therapeutic target at the maternal–fetal interface [71,79]. Nipocalimab is a fully human monoclonal antibody targeting FcRn, developed as a mechanism-based therapy for IgG-mediated diseases, including alloimmune conditions in pregnancy. By blocking FcRn, nipocalimab prevents IgG recycling and accelerates lysosomal degradation, reducing maternal IgG levels by up to 85% in healthy volunteers [80]. Consistent with these findings, maternal IgG levels decreased by approximately 85% from baseline in the UNITY phase 2 study of pregnancies at high risk for severe HDFN [81]. Concurrently, it decreases transplacental IgG transfer, thereby limiting fetal exposure to pathogenic antibodies. This dual mechanism targets an early step in disease pathogenesis, shifting management from treatment of established fetal injury to prevention of disease progression [71,80,82].

Clinical translation has been most extensively evaluated in HDFN [81]. In the phase 2 UNITY study, nipocalimab reduced disease severity in pregnancies at high risk for recurrent early-onset HDFN. The primary endpoint—live birth at or beyond 32 weeks without IUT—was achieved in 54% of cases (7 of 13), compared with historical rates ranging from 0% to 10%. No cases of hydrops were observed, and nearly half of pregnancies required no antenatal or postnatal transfusions. In those requiring intervention, the onset of severe fetal anemia was delayed, with the median gestational age at first IUT occurring at approximately 27 weeks compared with 20–22 weeks in historical cohorts. These outcomes were accompanied by marked pharmacodynamic effects, including an approximately 85% reduction in maternal IgG levels and a 4-to-32-fold decrease in alloantibody titers, supporting an association between FcRn inhibition and disease modification [81]. Phase 3 evaluation is ongoing in the AZALEA trial [83], a multinational, double-blind randomized study comparing nipocalimab with placebo in women aged 18–45 years enrolled at 13–18 weeks’ gestation with a history of severe HDFN. Eligibility requires clinically significant alloantibodies (e.g., anti-RhD, Rhc, RhC, RhE, or Kell) above critical thresholds (anti-Kell ≥ 4; others ≥ 16) and confirmed fetal antigen positivity, typically by cell-free fetal DNA testing.

FNAIT represents an equally compelling application, with a stronger emphasis on prevention. Unlike HDFN, where anemia can be detected and treated, fetal ICH in FNAIT often occurs in utero and may precede clinical detection, with substantial associated mortality and long-term neurologic morbidity [73,75]. FcRn blockade offers a strategy to reduce fetal exposure to antiplatelet antibodies before thrombocytopenia develops. This approach is being evaluated in two phase 3 trials. FREESIA-1 is a multinational, double-blind randomized placebo-controlled trial enrolling women aged 18–45 years at 13–18 weeks of gestation with prior FNAIT, maternal anti-Human Platelet Antigen (HPA)-1a antibodies, and an HPA-1a-positive fetus; pregnancies with prior severe hemorrhage or ICH are excluded [84]. FREESIA-3 is a similarly designed randomized trial comparing nipocalimab directly with IVIG, enrolling women aged 18–45 years at 13–18 weeks of gestation with prior FNAIT and maternal anti-HPA-1a and/or anti-HPA-5b antibodies with a corresponding antigen-positive fetus [85]. These studies aim to determine whether FcRn blockade can reduce severe thrombocytopenia and bleeding and potentially serve as an alternative to IVIG, reflecting a transition toward mechanism-targeted prophylaxis [84,85].

Despite its therapeutic promise, FcRn blockade raises important safety and ethical considerations related to maternal and neonatal immunologic effects. Given the role of FcRn in albumin homeostasis, serum albumin levels were also monitored as a potential safety signal. By design, nipocalimab reduces circulating maternal IgG and limits transplacental antibody transfer, resulting in lower neonatal IgG concentrations at birth [76,83]. In the UNITY follow-up analysis, median cord blood IgG levels were substantially reduced (175 mg/dL; range, 92–941), consistent with effective FcRn inhibition. Importantly, fetal and neonatal exposure to nipocalimab appeared minimal, with most cord blood and neonatal samples showing undetectable drug concentrations and only rare low-level detection below pharmacologically active thresholds. These findings indicate that reduced neonatal IgG reflects inhibition of maternal–fetal transfer rather than direct fetal immunosuppression [76].

Longitudinal follow-up provides further reassurance regarding immune recovery and clinical safety. Infant IgG levels reached a physiologic nadir between 4 and 24 weeks after birth and subsequently increased, with most infants achieving age-appropriate levels by 48 to 96 weeks. During this period, infections were largely consistent with typical early childhood patterns, with most events mild to moderate and resolving without complications; only a single severe infection (respiratory syncytial virus requiring hospitalization) was reported, with full recovery. Functional humoral immunity remained intact, with protective responses to routine vaccinations observed in most infants [76]. These findings suggest a transient reduction in passive immunity without sustained impairment of immune function. However, given limited sample sizes, continued evaluation in larger cohorts is necessary to define long-term safety [83,84,85].

### 3.5. Sirolimus for Fetal Lymphatic Malformations and Cardiac Rhabdomyoma

Fetal lymphatic malformations (LMs) and cardiac rhabdomyomas are distinct conditions characterized by abnormal tissue growth that may disrupt fetal physiology [86,87,88]. Cardiac rhabdomyomas, a major diagnostic feature of tuberous sclerosis complex (TSC), are often identified in mid-gestation and frequently regress after birth, but a subset is complicated by arrhythmia, outflow tract obstruction, hydrops, or fetal demise [89,90]. A recent systematic review of 400 fetuses reported prenatal regression in only 13% (with 58% postnatal regression) and substantial complication rates, including arrhythmia (13%), obstruction (16%), hydrops (10%), and fetal demise (12%), highlighting that the natural prenatal course is not uniformly benign [89]. LM demonstrate similarly variable behavior, ranging from stable lesions to rapidly enlarging masses associated with compression of adjacent structures, including airway compromise or hydrops [91]. In both, intervention is guided by physiologic compromise rather than diagnosis alone [89,92]. Both conditions share dysregulated mammalian target of rapamycin (mTOR) signaling [93]. In cardiac rhabdomyoma associated with TSC, pathogenic variants in TSC1 or TSC2 result in loss of tumor suppressor function and dysregulation of mTOR signaling, leading to abnormal cellular proliferation and hamartoma formation [90]. In lymphatic malformations, somatic activating mutations, most commonly involving PIK3CA, promote abnormal lymphangiogenesis through activation of the PI3K–AKT–mTOR pathway [93]. Despite distinct upstream mechanisms, both conditions depend on mTOR signaling to sustain abnormal tissue growth, providing a biologic rationale for prenatal mTOR inhibition.

Sirolimus, an mTOR inhibitor, has emerged as a transplacental, pathway-directed therapy to suppress abnormal tissue growth. Following maternal administration, the drug crosses the placenta and reaches the fetus, where it may reduce cellular proliferation, angiogenesis, and lymphangiogenesis, thereby limiting progression to clinically significant complications [93,94]. Transplacental transfer of sirolimus has been consistently demonstrated across fetal therapy reports, with measurable fetal exposure at clinically relevant levels. Cord blood sirolimus concentrations have been reported to be comparable to—or exceed—maternal levels at delivery, reaching 132–163% of maternal serum concentrations in some cases, supporting efficient placental transfer and biologically meaningful fetal exposure [95,96]. Similarly, a prenatal lymphatic malformation case report demonstrated fetal serum concentrations reaching approximately 20–28% of maternal levels, supporting clinically meaningful transplacental transfer [97].

The substantial variability in reported fetal sirolimus concentrations across published cases likely reflects differences in gestational age at sampling, maternal dosing regimens and serum concentrations, placental transport activity, maternal–fetal metabolism, and fetal drug distribution. The precise mechanism of placental transfer of sirolimus in humans remains incompletely defined. Although passive diffusion may contribute given its lipophilic macrolide structure, sirolimus is a relatively large molecule and its placental transfer is likely modulated by placental transport and metabolic systems [98]. Sirolimus is a known substrate of P-glycoprotein (MDR1/ABCB1) and is metabolized primarily through CYP3A pathways; therefore, placental efflux transporters and CYP3A-related metabolism may influence fetal exposure, although this has not been specifically characterized in fetal therapy studies [98]. The consistent detection of fetal sirolimus levels in reported cases supports clinically meaningful transplacental transfer, reinforcing its feasibility as a transplacental, mechanism-targeted therapy [95,96,97].

Clinical evidence for prenatal sirolimus therapy has evolved from initial case reports to small series demonstrating in utero regression of cardiac rhabdomyomas with confirmed fetal drug exposure [96,99]. Subsequent reports, including a structured three-fetus series, have consistently shown reduction in tumor size and improvement in complications such as outflow tract obstruction and cardiac dysfunction [89,96,100,101,102,103,104]. Similar findings have been reported in fetal lymphatic malformations, including one case in which lesion volume decreased from 357 cm^3^ to 50 cm^3^ with fetal sirolimus levels reaching approximately 30% of maternal concentrations, supporting effective transplacental exposure [97]. Clinical response is often rapid; however, tumor regrowth after treatment discontinuation suggests a suppressive rather than curative effect [99,100]. Overall, current evidence—derived primarily from case reports and small series—suggests promising but variable efficacy, likely reflecting underlying disease heterogeneity [97,105].

These limitations have motivated the development of prospective investigational protocols aimed at systematically evaluating prenatal sirolimus therapy in high-risk fetal lymphatic malformations. One such ongoing effort, the MaterPONS study (“Mom as a bridge to fetus”), is evaluating maternal sirolimus administration in singleton pregnancies complicated by severe cervicofacial LM with associated physiologic compromise. The proposed investigational framework includes carefully selected maternal and fetal eligibility criteria focusing on severe fetal disease while excluding major chromosomal abnormalities, multiple gestation, and significant maternal contraindications to sirolimus therapy. The study is designed to evaluate the feasibility and safety of prenatal sirolimus therapy, with primary outcomes focused on lesion progression, fetal airway or hydrops-related compromise, maternal tolerance, and longitudinal postnatal outcomes [106].

Safety data for prenatal sirolimus exposure remain limited. Preclinical animal studies and human experience from transplant populations suggest potential embryo–fetal toxicity, including fetal loss and impaired fetal growth, although a consistent teratogenic pattern has not been identified [107]. Interpretation of available human data remains challenging because of frequent polypharmacy and underlying maternal comorbidities in transplant cohorts. In fetal therapy reports, maternal tolerance has generally been acceptable, although fetal growth restriction has been observed in some cases [96,108]. These findings are biologically plausible given the role of mTOR signaling in placental nutrient sensing and fetal growth regulation [109]. Maternal adverse effects including hypertriglyceridemia requiring dose adjustment have also been reported, while known sirolimus-associated toxicities include mucositis, cytopenias, and hyperlipidemia [110]. Reported neonatal outcomes have generally been reassuring, although long-term follow-up data remain limited, supporting careful patient selection and monitoring.

In current practice, prenatal sirolimus is reserved for fetuses with progressive physiologic compromise, such as obstructive rhabdomyomas or high-risk LM with airway compromise. Prospective clinical trials, including the ongoing MaterPONS study, are now being initiated to evaluate the safety and feasibility of this approach. The results of these studies are highly anticipated and are expected to provide critical evidence to guide future clinical application.

## 4. Ethical Challenges

Fetal therapy has traditionally been limited to conditions where intervention could prevent fetal death or irreversible organ damage [111,112]. However, advances in prenatal diagnosis and molecular-targeted therapeutics have expanded this scope toward earlier, disease-modifying interventions. This shift challenges conventional ethical frameworks, moving beyond binary classifications such as “lethal” versus “nonlethal” disease [113,114]. Instead, ethical decision-making must incorporate anticipated biological impact, timing, and probability of benefit, requiring a proportional and context-sensitive approach [115,116].

### 4.1. The Maternal–Fetal Interface: Defining the Limits of Intervention

All fetal interventions are inherently maternal interventions. It is impossible to treat the fetus without exposing the pregnant individual to some level of risk, establishing maternal autonomy as the central ethical boundary [117]. A pregnant individual retains the right to accept or refuse treatment, even when refusal may result in fetal harm [118]. Fetal therapy introduces an asymmetry: benefits primarily accrue to the fetus, while risks are borne, at least partially, by the pregnant individual [114,119]. Ethical justification therefore requires that maternal risks remain proportionate to anticipated fetal benefit. While individuals may choose to accept risk for fetal benefit, such risks must remain reasonable and cannot be justified solely by potential fetal outcomes. [115,120]. This principle is illustrated using elexacaftor–tezacaftor–ivacaftor (ETI) during pregnancy, where continuation of therapy may provide substantial maternal benefit while simultaneously offering potential fetal benefit. Such situations highlight the ethical complexity of balancing maternal autonomy, maternal well-being, and uncertain fetal outcomes.

### 4.2. Acting Under Uncertainty

Uncertainty is intrinsic to molecular-targeted fetal therapies, which are often supported by limited human data and early-phase studies. Even when initial outcomes appear promising, benefits remain probabilistic and incompletely defined [113,120]. This creates a state of “precarious hope,” where patients may pursue intervention despite substantial uncertainty [120]. The prenatal use of risdiplam for spinal muscular atrophy exemplifies this challenge. In the reported case, treatment was initiated despite the absence of systematic human prenatal safety or efficacy data, requiring parents and clinicians to make decisions under conditions of profound uncertainty while balancing potential benefit against unknown risks. Clinicians must balance innovation with evidence, avoiding both premature adoption and unnecessary restriction [116,120]. A key challenge is distinguishing research from therapy. Labeling experimental interventions as treatment may contribute to therapeutic misconception, particularly in emotionally charged settings where patients feel compelled to pursue all possible options [119,120]. Ethically, uncertainty requires transparency and restraint. Interventions should not be offered when risks clearly outweigh benefits, but strict reliance on definitive evidence may delay access to potentially transformative therapies. A balanced approach includes careful evaluation of available data, explicit communication of uncertainty, and prioritization of structured research [117,120].

### 4.3. Preventive Versus Therapeutic Intervention

The expansion of fetal therapy into preventive domains represents a major ethical shift. Interventions are increasingly aimed at preventing long-term morbidity rather than rescuing a fetus in immediate distress. This raises a central question: when does the possibility of future disease justify present intervention [113,114]? Limiting fetal therapy to life-threatening conditions is no longer sufficient. Interventions aimed at improving long-term functional outcomes may be ethically acceptable when expected benefits outweigh risks [114]. In lower-risk scenarios, even modest benefit may justify intervention, whereas higher-risk procedures may be appropriate in severe disease without alternatives [115]. However, preventive approaches introduce the risk of overtreatment. Some fetuses may never develop severe disease yet may be exposed to unnecessary risks. This challenge is further complicated by the availability of pregnancy termination, which shapes both clinical decision-making and ethical considerations [111,117]. Nipocalimab provides a contemporary example of this ethical dilemma. In fetal and neonatal alloimmune thrombocytopenia (FNAIT), FcRn blockade is intended to prevent fetal intracranial hemorrhage before injury occurs, shifting the goal of intervention from treatment of established disease to prevention of a potentially devastating outcome.

### 4.4. Long-Term and Transgenerational Unknowns

Unlike many surgical interventions, molecular-targeted therapies may alter biological pathways with effects that extend throughout life. Short-term safety does not guarantee long-term safety, and adverse outcomes may not become apparent for decades. Additionally, gene- or pathway-targeted interventions raise concerns about potential effects on germline development and future generations [113]. A particularly difficult ethical scenario arises when intervention converts fetal death into survival with severe morbidity. Survival alone does not necessarily represent benefit, particularly when associated with significant long-term suffering. Ethical evaluation must therefore consider quality of life, functional outcomes, and the broader impact on the child and family, rather than survival alone [111,114,120].

### 4.5. Equity and Access

Advances in fetal therapy have not been accompanied by equitable access. Specialized care is concentrated in a limited number of centers, often located in urban academic settings. As a result, access is shaped by both geography and socioeconomic status. Over 40% of women of childbearing age in the United States live beyond practical access to specialized fetal therapy centers, in regions described as “fetal care deserts”. These areas are characterized by lower income, higher poverty rates, and reduced access to insurance and resources [121]. Disparities are multidimensional. Geographic distance limits physical access, while socioeconomic factors determine the ability to travel, relocate, and engage with care. Many patients may never reach specialized centers, limiting access to potentially beneficial interventions [121].

This creates a fundamental ethical tension between centralization, which improves outcomes through expertise and volume, and equitable access to care. Without coordinated referral systems and system-level solutions, these disparities are likely to persist [121,122].

### 4.6. Toward a Practical Ethical Framework

The ethical evaluation of in utero molecular-targeted therapies requires a structured and pragmatic approach. A proportionality-based framework provides a useful foundation, integrating fetal benefit, maternal risk, and broader clinical context [114,115]. Several core principles emerge. First, interventions should be offered only when anticipated benefits justify risks to both the fetus and the pregnant individual. Second, maternal autonomy must remain the central ethical boundary, supported by non-directive and transparent counseling. Third, uncertainty must be explicitly acknowledged, including limitations of current evidence and potential long-term risks. Fourth, wherever feasible, therapies should be delivered within structured research frameworks. When offered outside trials, systematic data collection and outcome reporting remain essential. Fifth, delivery of fetal therapy requires multidisciplinary expertise and institutional oversight to ensure safety and accountability. Finally, addressing inequities in access must be considered a core ethical responsibility. Expansion of specialized services, development of referral networks, and integration of emerging models of care are necessary to ensure that advances in fetal therapy translate into equitable clinical benefit.

## 5. Conclusions

In utero molecular-targeted drug therapies represent a transformative advance in fetal medicine, enabling earlier and more precise intervention for growing number of fetal conditions. The therapeutic approaches reviewed in this article span a broad spectrum of clinical maturity (Table 2). Nipocalimab currently has the most advanced evidence base, with ongoing phase 3 evaluation for prevention of severe alloimmune fetal disease. Prenatal enzyme replacement therapy is being evaluated in phase 1 clinical trials, while prenatal sirolimus therapy is entering prospective clinical investigation. In contrast, prenatal use of ETI and risdiplam remains supported primarily by case reports, case series, and emerging clinical experience. Together, these developments illustrate both the rapid progress and the uneven maturity of the current prenatal molecular therapeutics landscape.

Despite encouraging early results, important knowledge gaps remain. Future research priorities include prospective pharmacokinetic studies to better characterize maternal–fetal drug exposure, long-term neurodevelopmental and functional follow-up of exposed children, validation of biomarkers to guide patient selection and treatment monitoring, and systematic evaluation of maternal and fetal safety. Prospective pharmacokinetic and pharmacodynamic studies are particularly needed for emerging transplacental therapies such as risdiplam, sirolimus, and ETI to better define fetal drug exposure, target engagement, dose optimization, and safety monitoring.

This evolution also introduces complex ethical and regulatory challenges. None of the therapies reviewed currently holds regulatory approval specifically for prenatal use. To date, most clinical experience has been generated through compassionate-use frameworks, expanded access programs, investigator-initiated studies, or early-phase clinical trials. Continued collaboration among clinicians, investigators, industry partners, patient advocacy groups, and regulatory agencies will be necessary to establish evidence-based pathways toward broader clinical implementation.

As prenatal diagnosis continues to expand the spectrum of identifiable—and potentially treatable—fetal conditions [123], continued innovation must be accompanied by rigorous evaluation, transparent communication, and equitable implementation. Ultimately, the success of in utero molecular-targeted therapies will depend not only on scientific progress, but also on the ethical integrity with which these technologies are developed and applied, ensuring improved outcomes for both fetuses and their families.

## Figures and Tables

**Table 1:** Advantages and Disadvantages of the In Utero Approach for Molecular-Targeted Drug Therapies.

	**Underlying Rationale**	**Potential Implications**
**Advantages**		
Reduced therapeutic dose requirements	The small fetal size may allow therapeutic concentrations to be achieved with lower quantities of drug per recipient.	Improved feasibility and potentially reduced treatment costs.
Potential immune tolerance induction	The fetal immune system is relatively immature and enriched in regulatory T-cells.	Reduced anti-drug antibody formation and prolonged therapeutic activity.
Early disease modification	Treatment before disease progression becomes established.	Improved long-term organ function and disease modification.
**Disadvantages and Challenges**	**Underlying Rationale**	**Potential Implications**
Maternal drug exposure	Maternal treatment or fetal intervention may expose otherwise healthy pregnant individuals to risk.	Requires stringent maternal safety evaluation.
Off-target fetal effects	Therapeutic agents may affect unintended fetal tissues during development.	Potential developmental toxicity and unforeseen consequences.
Challenges in dose optimization	Placental transfer and fetal pharmacokinetics remain incompletely understood.	Difficulty establishing optimal dosing regimens.
Ethical considerations	Interventions are performed in a fetus unable to provide consent.	Complex ethical and regulatory oversight.
Limited long-term safety data	Most prenatal molecular therapies remain investigational.	Late adverse effects may not become apparent until later in life.

**Table 2:** Current and Emerging In Utero Molecular-Targeted Drug Therapies.

Disease/Indication	Therapy	Molecular Target/Mechanism	Route of Administration	Clinical Goal	Current Evidence	Status
Lysosomal storage diseases	Enzyme replacement therapy	Replacement of deficient lysosomal enzyme	Intravascular (umbilical vein)	Prevent irreversible prenatal organ injury	Case reports	Ongoing Phase 1 clinical trial ongoing (PEARL)
Spinal muscular atrophy	Risdiplam	SMN2 splicing modification/increased SMN protein	Maternal oral	Prevent prenatal motor neuron loss	Case reports	Clinical experience accumulating
Cystic fibrosis	Elexacaftor–tezacaftor–ivacaftor (ETI)	CFTR modulation	Maternal oral	Treat meconium ileus/preserve organ function	Case reports/case series	Clinical experience accumulating
Hemolytic disease of the fetus and newborn (HDFN)	Nipocalimab	FcRn blockade	Maternal intravenous	Reduce fetal anemia/delay or avoid IUT	Phase 2 clinical trial (UNITY)	Ongoing Phase 3 clinical trial (AZALEA)
Fetal and neonatal alloimmune thrombocytopenia (FNAIT)	Nipocalimab	FcRn blockade	Maternal intravenous	Prevent fetal thrombocytopenia/intracranial hemorrhage	Early clinical data	Ongoing Phase 3 clinical trial (FREESIA)
Lymphatic malformation	Sirolimus	mTOR pathway inhibition	Maternal oral	Reduce lesion growth/hydrops/physiologic compromise	Case reports	Phase 1 clinical trial Planned (MaterPONS)

Abbreviations: CFTR, cystic fibrosis transmembrane conductance regulator; FcRn, neonatal Fc receptor; IUT, intrauterine transfusion; mTOR, mechanistic target of rapamycin; SMN, survival motor neuron.

## Data Availability

No new data were created or analyzed in this study.
